# Association Between Circulating Proprotein Convertase Subtilisin/Kexin Type 9 and Major Adverse Cardiovascular Events, Stroke, and All-Cause Mortality: Systemic Review and Meta-Analysis

**DOI:** 10.3389/fcvm.2021.617249

**Published:** 2021-03-02

**Authors:** Yimo Zhou, Weiqi Chen, Meng Lu, Yongjun Wang

**Affiliations:** ^1^Department of Neurology, Beijing Tiantan Hospital, Capital Medical University, Beijing, China; ^2^China National Clinical Research Center for Neurological Diseases, Beijing, China; ^3^Center of Stroke, Beijing Institute for Brain Disorders, Beijing, China; ^4^Beijing Key Laboratory of Translational Medicine for Cerebrovascular Disease, Beijing, China; ^5^Department of Pharmacy, National Center of Cardiovascular Diseases, Fuwai Hospital, Chinese Academy of Medical Sciences and Peking Union Medical College, Beijing, China

**Keywords:** proprotein convertase subtilisin/kexin type 9, cardiovascular events, stroke, mortality, association

## Abstract

**Background:** Proprotein convertase subtilisin/kexin type 9 (PCSK9), a pivotal protein in low-density lipoprotein cholesterol metabolism, has been validated to be an established target for cardiovascular (CV) risk reduction. Nevertheless, prospective studies concerning the associations between circulating PCSK9 and the risk of CV events and mortality have yielded, so far, inconsistent results. Herein, we conducted a meta-analysis to evaluate the association systemically.

**Methods:** Pertinent studies were identified from PubMed, EMBASE, and Cochrane Library database through July 2020. Longitudinal studies investigating the value of circulating PCSK9 for predicting major adverse cardiovascular events (MACEs) or stroke or all-cause mortally with risk estimates and 95% confidence intervals (CI) were included in the analyses. Dose-response meta-analysis was also applied to evaluate circulating PCSK9 and risk of MACEs in this study.

**Results:** A total of 22 eligible cohorts comprising 28,319 participants from 20 eligible articles were finally included in the study. The pooled relative risk (RR) of MACEs for one standard deviation increase in baseline PCSK9 was 1.120 (95% CI, 1.056–1.189). When categorizing subjects into tertiles, the pooled RR for the highest tertile of baseline PCSK9 was 1.252 (95% CI, 1.104–1.420) compared with the lowest category. This positive association between PCSK9 level and risk of MACEs persisted in sensitivity and most of the subgroup analyses. Twelve studies were included in dose-response meta-analysis, and a linear association between PCSK9 concentration and risk of MACEs was observed (x2 test for non-linearity = 0.31, P non-linearity = 0.575). No significant correlation was found either on stroke or all-cause mortality.

**Conclusion:** This meta-analysis added further evidence that high circulating PCSK9 concentration significantly associated with increased risk of MACEs, and a linear dose-response association was observed. However, available data did not suggest significant association either on stroke or all-cause mortality. Additional well-designed studies are warranted to further investigate the correlations between PCSK9 concentration and stroke and mortality.

## Introduction

Proprotein convertase subtilisin/kexin type 9 (PCSK9), a circulating serine protease, has a fundamental role in low-density lipoprotein cholesterol (LDL-C) metabolism by enhancing the endosomal and lysosomal degradation of hepatic LDL-Receptor, thereby resulting in increased LDL-C concentration.

Over the past years, PCSK9 has been validated to be an established target for cholesterol-lowering therapies. Three randomized, double-blind, placebo-controlled cardiovascular (CV) outcome trials were completed and demonstrated that PCSK9 monoclonal antibodies significantly reduce plasma LDL-C level and major vascular events in subjects with high CV risk (1–3). The prespecified analyses designed to assess the effect of PCSK9 inhibitors on stroke demonstrated a reduction in risk of ischemic stroke (IS) without increasing hemorrhagic stroke, irrespective of baseline LDL-C and of prior IS history (4, 5). Moreover, emerging evidence has suggested that PCSK9 exerts pleiotropic effects beyond plasma LDL regulation, implying that PCSK9 might be a CV risk factor independent of LDL-C (6).

Circulating concentration of PCSK9 has attracted scientific interest as a biomarker for CV risk stratification. In recent years, mounting studies have explored the association between circulating PCSK9 and the risk of CV events; however, the results remained divergent. Werner et al. reported that elevated PCSK9 serum concentrations are correlated with CV events in patients with stable coronary artery disease (7). Nevertheless, Khoury et al. assessed the association and found that PCSK9 was inconsistently associated with CV events in two diabetes cohorts (8). In a large-scale primary prevention cohort, plasma levels of PCSK9 measured at baseline did not predict future CV events (9). Therefore, an updated meta-analysis concerning this topic was performed to improve statistical power and investigate the possible source of heterogeneity between published studies. Our meta-analysis differed from previously published meta-analyses by the inclusion of more recent studies, the inclusion of stroke as clinical outcome, exploring more potential aspects for heterogeneity sources. Accordingly, we conducted the current meta-analysis to add substantive new data and insights into the predictive ability of circulating PCSK9 level in terms of major adverse cardiovascular events (MACEs), stroke, and all-cause mortality from the eligible prospective studies.

## Methods

### Search Strategy and Selection Criteria

In accordance with recommendations of the Meta-analysis of Observational Studies in Epidemiology (MOOSE) group (10), we searched electronic databases (PubMed, Embase, and Cochrane) up to July 2020 using a combined MeSH heading and keyword search strategy; the query syntax of searching was shown in the Supplementary Materials (see search strategy). To avoid missing any relevant study, we also checked and manually searched the references of the included articles.

### Study Selection

Studies were deemed eligible if they: (1) included participants of any age across different countries; (2) had PCSK9 levels in plasma or serum at baseline as exposure of interest; (3) had clinical outcomes including MACEs and/or stroke and/or all-cause mortality; (4) were prospective cohort studies or nested case-control studies performed within prospective cohort with a minimum follow-up of 1 year; (5)were full-text publications; (6) had a multivariable-adjusted relative risk (RR) or hazard ratio (HR) or odds ratio (OR) and the corresponding 95% confidence interval (CI) or provision of available information to calculate them. MACEs were defined as composite outcomes, including fatal and non-fatal coronary artery disease (CAD), fatal and non-fatal stroke, and heart failure. In order to better evaluate the causality between PCSK9 concentration and the clinical outcomes, we included only prospective cohort studies or prospective nested case-control studies.

### Data Extraction and Quality Assessment

Two reviewers (YZ and WC) independently searched, selected studies and extracted data. The disagreement between the two reviewers was resolved by consensus. The following data were extracted from each study: the last name of the first author, year of publication, country of study, type and amount of participants, study type, the proportion of men, mean age, duration of follow-up, number of outcome events, CV risk status, the measurement method of PCSK9, mean or median concentration of PCSK9, sample source, adjusted confounding factors, statin use, the history of family hypercholesterolemia (FH), and the most fully adjusted HRs or RRs or ORs with 95% CIs of circulating PCSK9.

Using the Newcastle-Ottawa Scale (NOS), the quality of the included studies was assessed (11). Each study was evaluated on three broad criteria: (1) subject selection; (2) comparability of the subjects; and (3) ascertainment of the outcome or exposure. Two reviewers independently evaluated the quality of each study. Disagreements were resolved through discussion to reach a consensus. A star system ranging between zero to nine stars is used to allow a semi-quantitative assessment of study quality. Studies which scored seven points or more were considered high quality.

### Statistical Analysis

The risk estimates of the association between PCSK9 and the outcomes of interest in each study were reported as a HR, RR, or OR with 95% CI. Risk estimates adjusted for the maximum number of covariates were pooled across studies for inclusion in the meta-analyses. In this meta-analysis, all associations were estimated as RRs and 95% CIs. HRs were approximately considered as RRs, which have been commonly used in previous studies (12). ORs were transformed into RRs using the formula RR = OR/[(1−P0) + (P0 × OR)] where P0 is the incidence of the outcome of interest in the non-exposed group, and OR was considered equivalent to the RR in cohort studies if the value of P0 was small (13). Both continuous (per one unit or one standard derivation (SD) increase) and categorical (tertiles or quartiles) variables of circulating PCSK9 were used in the included literature. In order to acquire a consistent comparison of the results, we transformed the RR of each study to standard risk estimates for 1-SD increase in PCSK9 levels, as well as for the highest tertile vs. lowest one for PCSK9 distribution using methods described previously (14, 15); briefly, these transformed estimates were calculated by multiplying the log RR and the upper and lower CIs for 1-SD increase with a scaling factor (2.18 for tertiles, and 2.54 for quartiles). The scaling methods assume that the PCSK9 is log-normally distributed and a log-linear association with the outcome. For normally transformed PCSK9, RR reported per unit were first converted to 1-SD increase, using the study-specific SD and then to tertiles.

Heterogeneity of RRs was evaluated by calculating the Cochrane *Q* statistic (*P* < 0.10 was deemed to be statistically significant) and the *I*^2^ statistic (low heterogeneity, *I*^2^ ≤ 50%; moderate heterogeneity, 50% < *I*^2^ < 75%; high heterogeneity, *I*^2^ ≥ 75%) (16). We pooled the RRs of the outcomes of interest using the random effects model (*I*^2^ > 50%, the DerSimonian-Laird method) or fixed effects model (*I*^2^ ≤ 50%, the Mantel-Haenszel method) as appropriate. To test the robustness of the pooled results, sensitivity analyses were conducted by leave-one-out method in each turn to investigate the influence of every single study on the overall risk estimate (17), and by excluding two nested case-control studies.

To assess the potential sources of heterogeneity, subgroup analyses which sorted by published year, mean age at baseline, sample size, CV risk, percentage of the history of FH, sample source, percentage of statin use, PCSK9 level at baseline, the assays for PCSK9 measurement, and degree of adjustment were performed. High CV risk cohort referred to participants in the studies with established CVD or known CVD risk factors (such as chronic renal disease, atrial fibrillation, type 2 diabetes, familial hypercholesterolemia, and hemodialysis) and low CV risk cohort to apparently healthy participants at baseline. Mean/median level of PCSK9 at baseline for included studies were extracted, and 258 ng/mL, the median for the 21 cohorts, was used as the cut-off point. A univariate meta-regression with restricted maximum likelihood was performed to measure if pooled RR significantly differed between each stratum analyzed.

Additionally, a dose-response meta-analysis was further conducted to determine a potential curvilinear (non-linear) or linear association between circulating PCSK9 and risk of MACEs. We used the two-stage generalized least-squares trend (GLST) estimation method proposed by Greenland and Longnecker to estimate the study-specific slope lines first and then derive an overall average slope (18, 19). This method requires the cases and cohort size/control subjects of each category and the risk estimate with its variance estimate for at least three quantitative exposure categories to be known. We excluded the studies without the aforementioned values required for the dose-response meta-analysis or without sufficient data for deriving them. The dosage value assigned to each stratum of PCSK9 was the median or mean in each category provided by the original article. In terms of the studies not containing median/mean, the midpoint was used for closed category and the same amplitude as the adjacent category for the open-ended highest or lowest category. A restricted cubic spline with three knots (two spline transformations) was first created, and then a *P* for non-linearity was calculated to detect potential departure from a linear trend by testing the coefficient of the second spline equal to zero. In the presence of substantial linear trends (*P*_non−linearity_ > 0.05), a linear model was conducted to achieve the association between circulating PCSK9 and the risk of MACEs by using the method of two-stage GLST (19).

The possibility of publication bias of the outcome of MACEs was assessed graphically by funnel plots and quantitatively by Begg's rank correlation test and Egger's linear regression test (20, 21). Where asymmetry of the plot was found, a contour-enhanced funnel with the trim and fill method was further applied to differentiate asymmetry due to publication bias from that due to other factors (22). Statistical analysis was performed with STATA package, version 15.1 for Mac (StataCorp, College Station, TX, USA). *P* < 0.05 was considered statistically significant, except where otherwise specified.

## Results

### Literature Search and Study Characteristics

**Figure 1:**
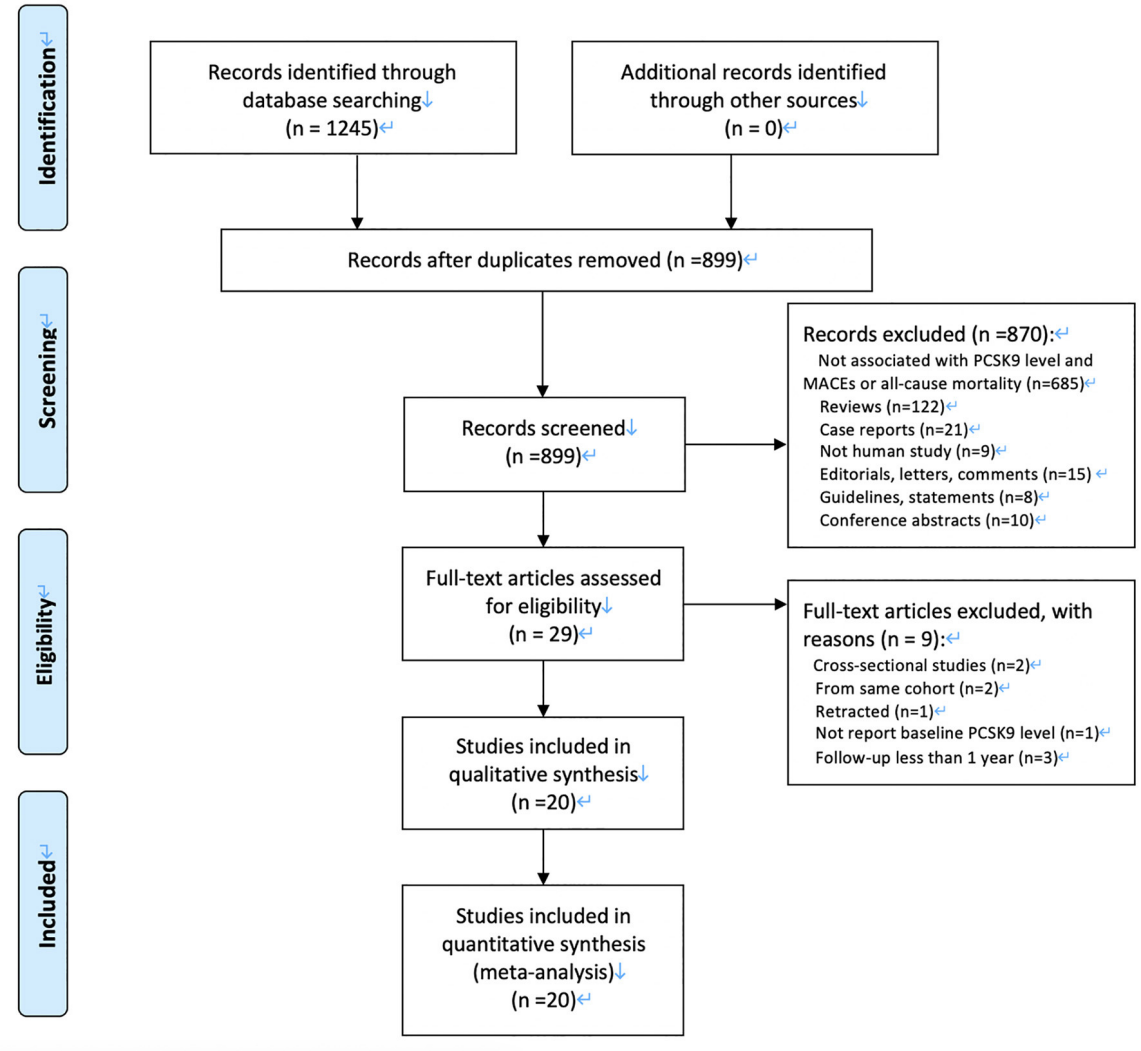
PRISMA flow diagram for the study selection procedure.

Our initial search returned 1,245 articles. After we screened titles and abstracts, 29 articles were qualified for full-text evaluation. After full-text review, nine studies were excluded, and 22 eligible cohorts from 20 eligible articles were finally included for meta-analysis (7–9, 23–39). Figure 1 demonstrates a flowchart for the study selection.

Table 1 summarizes the main characteristics of the included studies. Among the 20 articles, 19 articles were included in the analysis of MACEs, and six (8, 25, 26, 31, 34, 35) were in the all-cause mortality. The publication period of these articles ranged from 2014 to 2020, and the sample size of each study range from 151 to 5,307, with a total of 28,319 participants. Two studies were nested case-control studies, and the others were prospective cohort studies. According to quality assessment criteria, all but one studies were graded as high quality. A total of 12 studies reported risk estimates according to tertiles, two studies according to quartiles, and 16 studies according to continuous levels of PCSK9.

**Table 1:** Main characteristics of the included article.

**Study (first author, year)**	**Country**	**Study design**	**Population**	**Age (years, mean or median), male (%)**	**Follow-up (years, mean or median)**	**Number of outcome events**	**Measurement method**	**PCSK9 concentration (mean ±*SD* or median and IQR, ng/mL)**	**Degree of adjustment**	**NOS scale**
Wernar et al. (7)	Germany	Prospective cohort	504 patients with stable CAD	68, 83.3	2	96 ACS, 199 unplanned revascularizations, 52 CV death or non-fatal MI	ELISA using the CircuLex Human PCSK ELISA Kit (CY-8079, CycLex, Japan) in serum	548 (422–676) in 486 subjects with statin treatment; 454 (383–580) in 28 subjects without statin treatment.	+++++	8
Zhu et al. (36)	Canada	Prospective cohort	1,527 middle-aged firefighters without previously experienced cases of CAD, CVD, or peripheral vascular disease	49.4,100	7.2	111 events included 21 non-fatal MIs and 41 revascularizations, 12 cerebrovascular events, and one peripheral vascular event.	ELISA using the R&D Systems^®^ Quantikine Elisa Kit (R&D Systems^®^, 614 McKinley Place NE, Minneapolis MN, USA) in serum	286 (231–355)	+++++	8
Li et al. (37)	China	Prospective cohort	616 non-treated patients with stable CAD	57.88,72	1.4	72 (11.9%) patients presented with at least one MACE (four cardiac deaths, four non-fatal strokes, six MIs, 28 revascularizations, and 30 unstable anginas).	Serum PCSK9 concentrations were measured using a high-sensitivity, quantitative sandwich enzyme immunoassay (Quantikine ELISA, R&D Systems Europe Ltd, Uppsala, Sweden)	230.1 (190.5–277.8)	+++	8
Gencer et al. (35)	Switzerland	Prospective cohort	2,030 ACS patients	63.6, 78.9	1	90 all-cause death. Sixty-eight recurrent MI, 25 stroke, 66 coronary revascularizations.	Colorimetric ELISA from R&D Systems (Minneapolis, MN, USA) in serum	323 ± 134	+++++	9
Leander et al. (32)	Sweden	Prospective cohort	4,232 60-year-old men and women	60, 62.7	15	485 CVD events: fatal or non-fatal MI, angina pectoris, chronic ischemic heart disease, sudden cardiac death, and fatal or non-fatal IS.	ELISA was developed in-house by Eli Lilly laboratories in serum.	94.3 (73.1–122.3)	++++	8
Ridker et al. (9)	USA	Nested case-control	716 healthy American women	63, 0	17	358 participants developed first ever CVEs (MI, thromboembolic stroke, or CV death)	PCSK9 levels were measured using a commercial assay (Quantikine Human Proprotein Convertase 9/PCSK9 Immunoassay, R&D Systems)	304.4 (252.9–365.9) in the case, 299.7 (252.9–358.8) in the controls	++	8
Rogacev et al. (30)	Germany	Prospective cohort	443 patients (CARE FOR HOMe cohort) and 1,450 patients (LURIC cohort) with GFR between 90 and 15 ml/min/1.73 m^2^	67.7, 60 and 67.0, 64	3.0 and 10.0	91 patients with primary end-point (acute MI; surgical or interventional coronary/cerebrovascular/ peripheral-arterial revascularization; stroke with symptoms > 24 h, amputation above the ankle; or death of any cause) and 335 CV deaths	ELISA using the Circulex Human PCSK9 ELISA Kit (CY-8079, CycLex, Japan) and the Quantikine Human PCSK9 sandwich immunoassay (R&D, Minneapolis, MN, USA)	343 (270–413) and 208 (161–264)	++++ and +++	8
Laugsand et al. (27)	Norway	Nested case-control	5,307 residents aged 20 years or older without history of MI, angina pectoris, or stroke	66.2, 62.9	11.3	1,587 participants were diagnosed with a first incident MI	Serum levels of PCSK9 were measured in duplicate by enzyme immune assay with antibodies obtained from R&D Systems (Minneapolis, Minnesota)	123 ± 53	+++	7
Pastori et al. (29)	Italy	Prospective cohort	907 patients with atrial fibrillation	73.5, 57	3.4	179 CVE: 39 fatal or non-fatal MIs, 20 cardiac revascularizations, 72 CV deaths, and 48 CVEs.	Plasma levels of PCSK9 were measured by a commercial ELISA.	1.2 (0.9–1.97)	+++	8
Silbernagel et al. (25)	Austria	Prospective cohort	2,139 patients with or without CVD referred to coronary angiography in Cardiology unit	62.6, 68.7	10.1	674 (31.5%) study participants died from any cause	Total PCSK9 was measured using the Quantikine Human PCSK9 sandwich immunoassay (R&D, Minneapolis, Minnesota, USA)	220 ± 82	++++	7
Eisenga et al. (26)	Netherlands	Prospective cohort	453 renal transplant recipients	51, 56	10	123 all-cause death	Serum PCSK9 was assessed by PCSK9 dual monoclonal antibody sandwich ELISA, with minor modifications	107.1 ± 43.4	+++++	7
Navarese et al. (24)	Germany	Prospective cohort	333 ACS patients receiving prasugrel or ticagrelor and undergoing PCI	57, 79.9	1	13 patients (22.03%) in the upper PCSK9 tertile experienced a clinical MACEs (CV death, MI, unstable angina, stent thrombosis, repeat revascularization and IS), compared with 2 (3.39%) in the lower PCSK9 tertile	ELISA using the CircuLex Human PCSK ELISA Kit (Medical and Biological Laboratories Co., Ltd., Japan) in serum	394.80	++	7
Khoury et al. (8)	France	Prospective cohort	2,911 unrelated French patients in high CV risk men and women with type 2 diabetes selected on the basis of persistent microalbuminuria (UAE = 20–200 mg/L) or macroalbuminuria (UAE > 200 mg/L) without renal failure (plasma creatinine <150 μmol/L) (DIABHYCAR cohort) and 1,468 patients with type 2 diabetes (SURDIAGENE cohort)	66, 73 and 65, 58	4.5 and 7.4	647 all CVEs (MI, stroke, TIA and heart failure leading to hospital admission, coronary/peripheral angioplasty or bypass, CV death); 175 stroke/TIA; 196 CV death and 616 all CVEs (MI, stroke, TIA and heart failure leading to hospital admission, coronary/peripheral angioplasty or bypass, CV death); 89 stroke/TIA; 307 CV death.	Using a commercial ELISA kit (Human Proprotein Convertase 9/PCSK9 Duoset catalog no. DY3888; R&D Systems, Minneapolis, Minnesota) to measure PCSK9 level in plasma	40.0 (28.5–54.8), 43.8 ± 21.9 and 87.8 (66.4–113.7), 92.0 ± 37.4	+++++ and +++++	8
Gao et al. (38)	China	Prospective cohort	1,646 patients with AMI	61, 74.8	1	37 cardiac death, 27 non-fatal acute MI, 82 coronary revascularizations, and 10 IS	PCSK9 in plasma was measured *via* colorimetric ELISA (Human Proprotein Convertase 9/PCSK9 Immunoassay, DPC900, R&D Systems).	279 (230–349)	+++	7
Rasmussen et al. (39)	Denmark	Prospective cohort	151 kidney transplantation candidates with end-stage renal disease	57.7, 68.2	3.7	32 MACEs (cardiac arrest with successful resuscitation; ST-elevation MI; non-ST-elevation MI and/or revascularization) and 29 deaths	ELISA using a human PCSK9 Quantikine ELISA kit (DPC900) from R&D Systems (MN, USA) in plasma	258 ± 16	++	8
Zhang et al. (23)	China	Prospective cohort	281 patients with definite time of onset of acute MI and who underwent primary PCI within 24 h of onset	Male (58.53); female (68.64), 78.3	1	18 MACEs (cardiac death, stroke, recurrent acute MI, and target vessel revascularization)	Plasma PCSK9 concentration were measured in the stored plasma samples using a quantitative sandwich enzyme immunoassay ELISA (catalog number Circulex CY-8079; CycLex Co., Ltd., Japan),	283.8 (227.7–393.3)	+++++	7
Cao et al. (1, 28)	China	Prospective cohort	338 patients with heterozygous FH hospitalized because of angina-like chest	49.38, 58.6	3	33 FH participants developed a MACE (nine died, two developed MI, six had stroke, four experienced re-admission due to unstable angina pectoris and 12 underwent PCI or CABG)	Plasma PCSK9 concentrations were determined by a commercial sandwich enzyme immunoassay (Quantikine ELISA, R&D System Europe Ltd, Minneapolis, USA)	322.18 (249.97–396.24)	+++++	7
Cao et al. (2, 33)	China	Prospective cohort	249 heterozygous FH patients with angiographic proven CAD who had experienced a first CVE	50.23, 56.2	3.6	29 recurrent CVEs (three MI, four stroke, 12 revascularization, 10 cardiac death)	Plasma PCSK9 concentrations were determined using a high-sensitivity, quantitative sandwich enzyme immunoassay (Quantikine ELISA, R & D Systems Europe Ltd)	363.9	+++++	9
Strålberg et al. (34)	Sweden	Prospective cohort	265 patients starting hemodialysis	66, 59	3	134 deaths: CV (*n* = 67, 50%), withdrawal of dialysis (*n* = 23, 17%), infection (*n* =16, 12%), malignancy (*n* = 9, 7%), other (*n* = 15, 11%), and unknown (*n* = 4, 3%)	A sandwich ELISA from R&D Systems (Abingdon, UK) was used for the quantitative determination of Human PSCK9 in serum	318 ± 170	+++	8
Hwang et al. (31)	Korea	Prospective cohort	353 hemodialysis patients	62.2, 67.1	2.4	30 deaths and 60 CVEs [CAD (CABG, percutaneous intervention, or MI), heart failure, ventricular arrhythmia, cardiac arrest, cerebral infarction, and peripheral vascular occlusive diseases requiring revascularization or surgical intervention]	The ELISA method was performed using Magnetic Luminex^®^ Screening Assay multiplex kits (R&D Systems, Inc., Minneapolis, MN, USA) in plasma	36.6 ± 20.3	+++++	6

*++, plus traditional CV risk factors; +++, plus statin use or metabolic biomarkers; ++++, plus statin use, and metabolic biomarkers; +++++, plus related clinical measurement or other medication treatment or genetic mutations*.

*ACS, acute coronary syndrome; CAD, coronary artery disease; CV, cardiovascular; CVD, cardiovascular disease; CVE: cardiovascular event; MI, myocardial infarction; IS, ischemic stroke; TIA, transient ischemic attack; ELISA, enzyme-linked immunosorbent assay; UAE, urinary albumin excretion; FH, family hypercholesterolemia; PCI, percutaneous coronary intervention; CABG, coronary artery bypass grafting; DM, diabetes mellitus; CARE FOR HOMe, Cardiovascular and Renal Outcome in CKD 2–4 Patients—The Forth Homburg evaluation; LURIC, Ludwigshafen Risk and Cardiovascular Health Study; DIABHYCAR, Non-Insulin Dependent Diabetes, Hypertension, Microalbuminuria or Proteinuria, Cardiovascular Events and Ramipril; SURDIAGENE, Survie, Diabète de type 2 et Génétique; NOS, Newcastle-Ottawa Scale: PCSK9, proprotein convertase subtilisin/kexin type 9*.

### Association Between PCSK9 and MACEs

#### PCSK9 as a Continuous Variable

**Figure 2:**
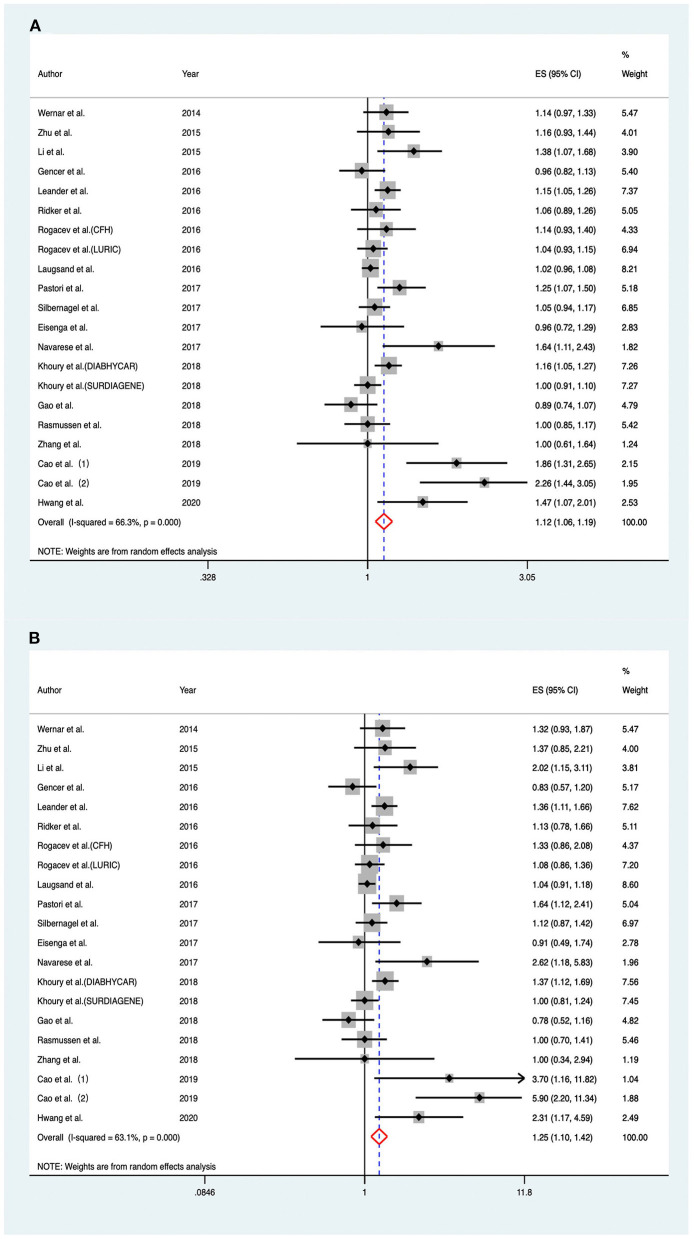
Associations between circulating proprotein convertase subtilisin/kexin type 9 and risk of major adverse cardiovascular events. **(A)** Per one standard derivation increase in baseline proprotein convertase subtilisin/kexin type 9 levels, **(B)** top vs. bottom tertile of baseline proprotein convertase subtilisin/kexin type 9. CFH indicates Cardiovascular and Renal Outcome in CKD 2–4 Patients—The Forth Homburg evaluation; LURIC, Ludwigshafen Risk and Cardiovascular Health Study; DIABHYCAR, Non-Insulin Dependent Diabetes, Hypertension, Microalbuminuria or Proteinuria, Cardiovascular Events and Ramipril; SURDIAGENE, Survie, Diabète de type 2 et Génétique; ES, effect size; CI, confidence intervals.

As shown in Figure 2A, RRs of the risk of MACEs for an increase in baseline PCSK9 by 1-SD varied from 0.89 to 2.26 across different cohorts, and a significantly positive association was found when pooling the risk estimate in a random-effect model (RR 1.120; 95% CI: 1.056–1.189; *P* < 0.001), with moderate heterogeneity across studies (*I*^2^ = 66.30%; *P*_heterogeneity_ < 0.001).

**Figure 3:**
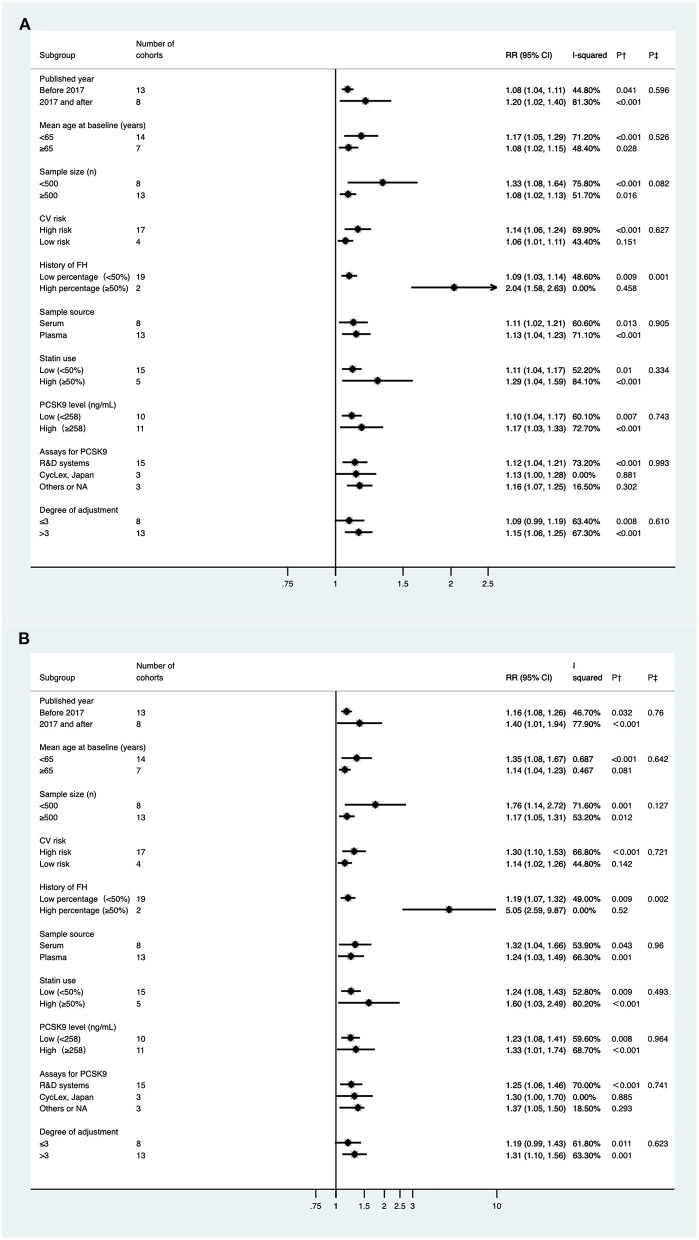
Subgroup analyses for circulating proprotein convertase subtilisin/kexin type 9 and the risk of major adverse cardiovascular events. **(A)** Per one standard derivation increase in baseline PCSK9 levels, **(B)** top vs. bottom tertile of baseline PCSK9. P^†^, for heterogeneity within each subgroup with *Q*-test. P^‡^, for difference between subgroups with meta-regression analysis. FH, family hypercholesterolemia; PCSK9, Proprotein convertase subtilisin/kexin type 9; CV, cardiovascular; RR, relative risk; CI: confidence intervals.

Considering the aforementioned moderate heterogeneity between the included studies, we further conducted subgroup analyses based on potential clinical relevance (Figure 3A). The positive association between PCSK9 level and risk of MACEs persisted in most of the subgroup analyses. The association was much stronger in studies with a high percentage of FH (RR 2.038; 95% CI: 1.576–2.634; *P* < 0.001) than those with a low percentage (RR 1.085; 95% CI: 1.035–1.138; *P* = 0.001); the heterogeneity was also reduced, indicating that the source of heterogeneity appeared to be contributed by the medical history of FH. High baseline PCSK9 level was only significantly associated with increased MACEs in studies with a higher degree of cofounder adjustment (RR 1.149; 95% CI: 1.057–1.248; *P* = 0.001) but not in those with a lower degree (RR 1.085; 95% CI: 0.994–1.189; *P* = 0.067).

For sensitivity analysis, exclusion of any single study did not immensely alter the combined risk estimate (Supplementary Figure 1A). After excluding two nested case-control studies, the combined RR did not substantial change (RR 1.139; 95% CI: 1.064–1.220; *P* < 0.001; *I*^2^ = 66.90%; *P*_heterogeneity_ < 0.001).

#### PCSK9 as a Category Variable

Considering the fact that most of the included articles reported circulating PCSK9 as tertiles, we also compared individuals within the top tertile with the bottom tertile of circulating PCSK9 levels at baseline. Overall, there was a significant association between the highest PCSK9 tertile and the risk of MACEs (RR, 1.252; 95% CI: 1.104–1.420) (Figure 2B), with moderate heterogeneity across studies (*I*^2^ = 63.10%; *P*_heterogeneity_ < 0.001).

Subgroup and sensitivity analyses for category PCSK9 achieved similar results to the analyses mentioned above for PSCK9 per 1-SD increase (Figure 3B, Supplementary Figure 1B); Yet, when pooling the risk estimate for cohorts using ELISA (CycLex, Japan) independently, the correlation between baseline PCSK9 and MACEs lost significance (RR, 1.302; 95% CI: 0.998–1.697).

#### Dose–Response Meta-Analysis

**Figure 4:**
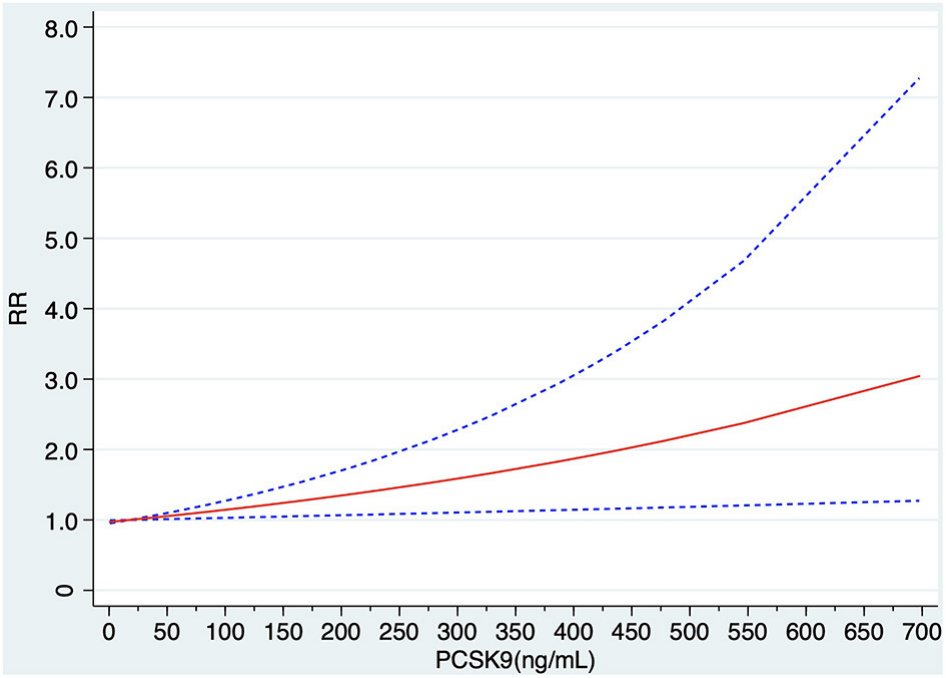
Linear dose–response of relationship between circulating PCSK9 concentration and risk MACEs. RR, relative risk.

Among the 19 articles concerning PCSK9 and MACEs, seven articles were excluded because of lack of cases or cohort size or the risk estimate of each category, and 12 articles were finally involved in the dose-response meta-analysis (7–9, 25, 27–29, 31–33, 35, 38). Using a restricted cubic spline model, no significantly curvilinear (non-linear) association was observed through a test for non-linearity (x2 test for non-linearity = 0.31, P non-linearity = 0.575). The linear dose–response curve demonstrated that the risk of MACEs increased slightly with elevation of PCSK9 concentration (Figure 4).

### Association Between PCSK9 and Stroke

In total, only two studies (8, 28), including three cohorts reported results on stroke (Supplementary Figure 2). Baseline PCSK9 could not significantly predict stroke when combining risk estimate by random effect models both for per 1-SD increase (RR, 1.022; 95% CI: 0.771–1.354; *I*^2^ = 57.6%, *P*_heterogeneity_ = 0.095) and for the highest tertile vs. the lowest tertile (RR, 1.051; 95% CI: 0.567–1.918; *I*^2^ = 63.1%, *P*_heterogeneity_ < 0.001).

### Association Between PCSK9 and All-Cause Mortality

**Figure 5:**
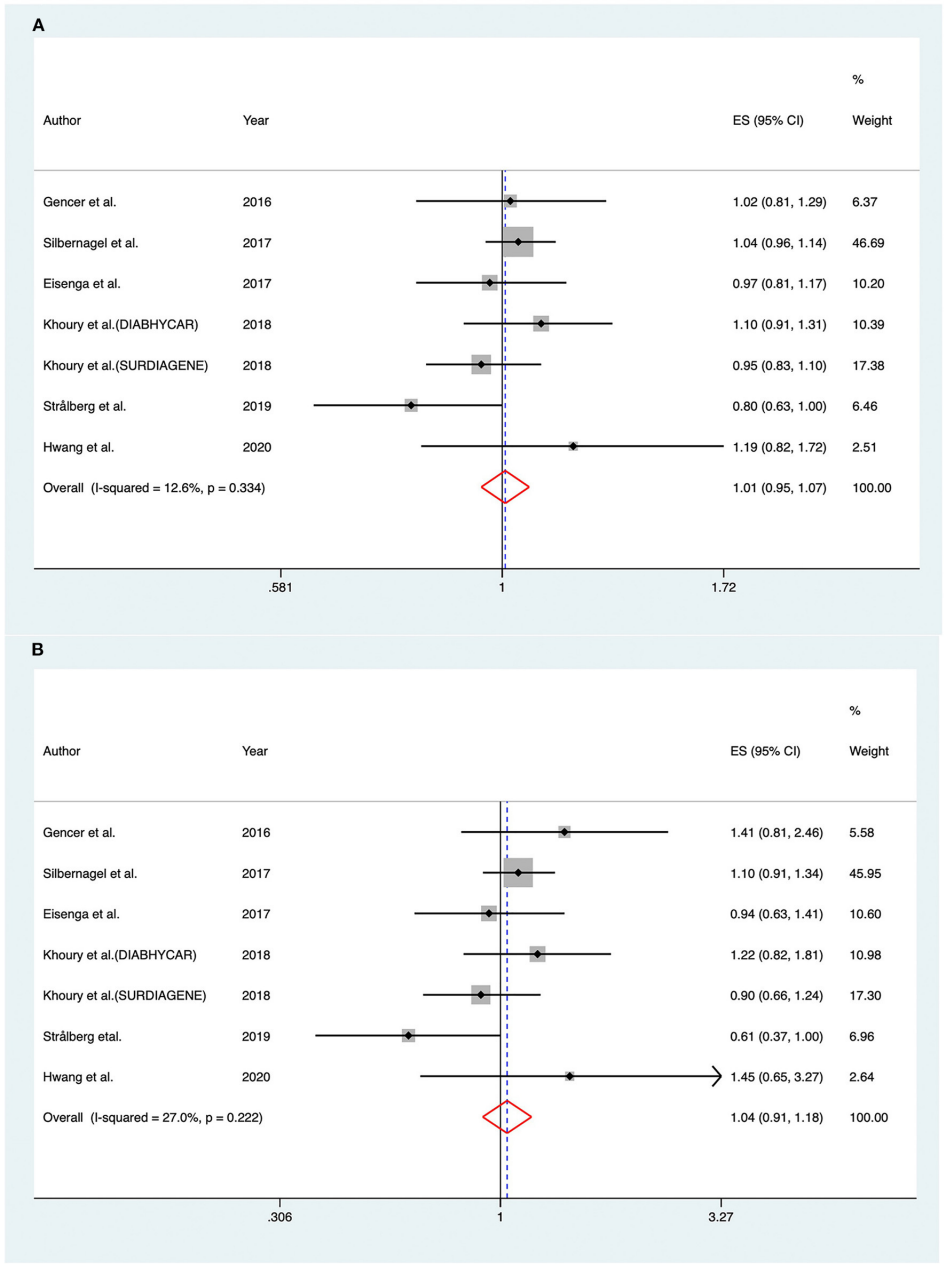
Associations between circulating proprotein convertase subtilisin/kexin type 9 and risk of all-cause mortality. **(A)** Per one standard derivation increase in baseline proprotein convertase subtilisin/kexin type 9 levels, **(B)** top vs. bottom tertile of baseline proprotein convertase subtilisin/kexin type 9. DIABHYCAR indicates Non-Insulin Dependent Diabetes, Hypertension, Microalbuminuria or Proteinuria, Cardiovascular Events, and Ramipril; SURDIAGENE, Survie, Diabète de type 2 et Génétique; ES, effect size; CI, confidence intervals.

The association between PCSK9 levels and risk of all-cause mortality was investigated in six studies (8, 25, 26, 31, 34, 35). The pooled RR of all-cause mortality in fixed-effect model for 1-SD increase in baseline PCSK9 was (RR 1.007; 95% CI: 0.950–1.068; *I*^2^ = 12.60%, *P*_heterogeneity_ = 0.334) (Figure 5A). For subjects distributed in the highest tertile of baseline PCSK9, the pooled RR was (RR 1.036; 95% CI: 0.909–1.181; *I*^2^ = 27.00%, *P*_heterogeneity_ = 0.222) (Figure 5B).

### Small-Study Effect and Publication Bias

**Figure 6:**
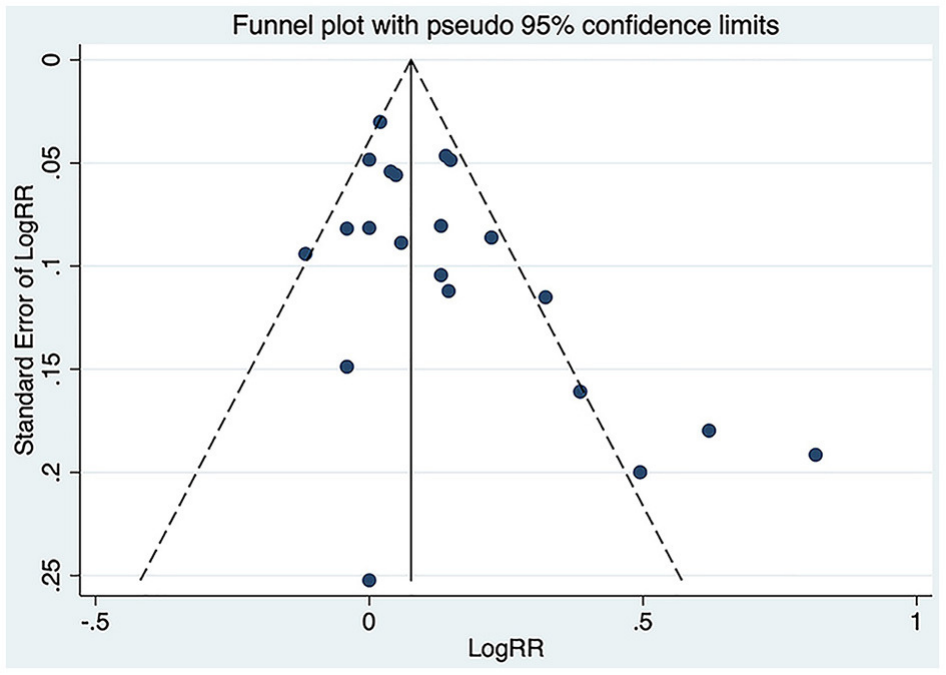
Funnel plot of the association between circulating proprotein convertase subtilisin/kexin type 9 concentration and major adverse cardiovascular events. RR, relative risk.

The funnel plot for the correlation between PCSK9 and MACEs showed asymmetry (small-study effect) at its bottom (Figure 6), which was confirmed by Begg's and Egger's test (*P* = 0.020, 0.016, respectively).

**Figure 7:**
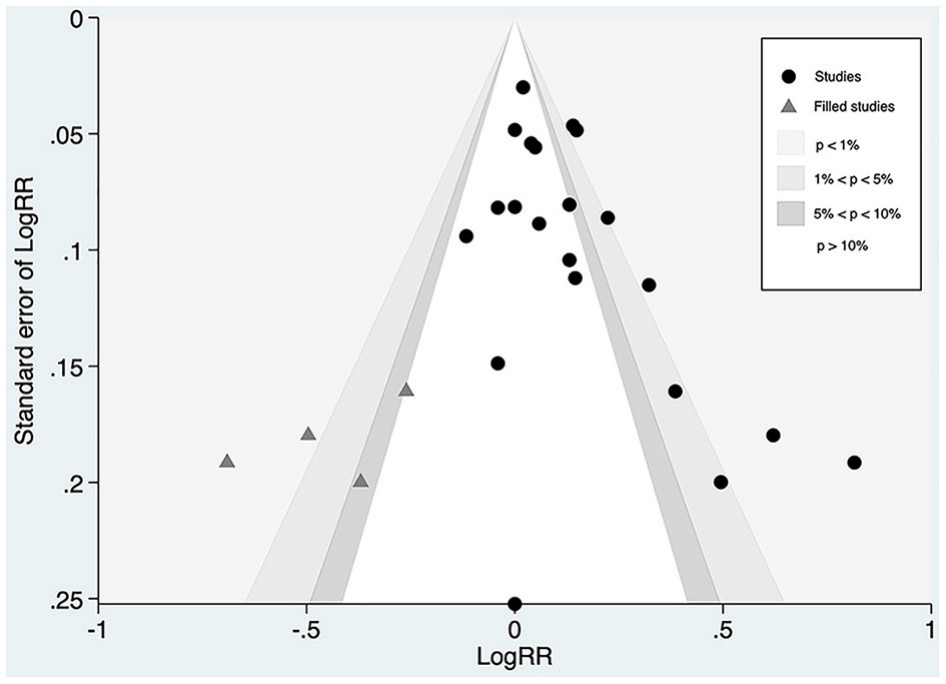
Contour-funnel plot of circulating proprotein convertase subtilisin/kexin type 9 concentration and major adverse cardiovascular events with “filled” studies estimated from the trim-and-fill method plotted. RR, relative risk.

Whereas, the contour-enhanced funnel plot with four filled studies estimated from the trim-and-fill method plotted (Figure 7) demonstrated that the “missing” studies were expected to lie in areas of high statistical significance, indicating that the small-study effect may not be due to publication bias.

## Discussion

Our study is an updated meta-analysis investigating the predictive role of circulating PCSK9 with clinical outcomes. In the present meta-analysis, 20 published articles, namely, 19 studies on MACEs, three studies on stroke, and six studies on all-cause mortality, involving a total of 28,319 participants were included. The result indicated that the PCSK9 level is an independent predictive marker for MACEs with a 25% increased risk while compared with the lowest tertile, and per unit of SD change in baseline PCSK9 corresponds to an increase of 12%. A dose-response meta-analysis between circulating PCSK9 concentration and MACEs risk was conducted further, and a linear dose-response relationship was observed. However, a significant association either with stroke or all-cause mortality was not suggested in the study.

Since substantial heterogeneity was observed across studies for MACEs, subgroup and meta-regression analysis were employed to get more reliable pooled risk estimates. Despite consistent results found in most of the subgroup analyses, heterogeneity could be partially explained by the percentage of FH history in the study (i.e., more robust association in studies with a high percentage of FH history).

The association between circulating PCSK9 level and CV risk has been investigated in three previous meta-analyses. In line with the findings reported by Vlachopoulos et al., Qiu et al., and Xiao et al., circulating PCSK9 is associated with increased CV risk when compared the highest with the lowest category (40–42). PCSK9 increases LDL concentration by enhancing LDLR degradation and preventing LDLR recirculation back to the cell membrane (43). Apart from regulating cholesterol metabolism by directly targeting LDLR, experimental studies suggested that PCSK9 could affect vascular biology and accelerate the progression of atherosclerosis *via* other mechanisms (44). Increased expression of PCSK9 is related to oxidized LDL-induced apoptosis in endothelial cell, which may give rise to subsequent endothelial dysfunction and pathogenesis of atherosclerosis (45). It is noteworthy that PCSK9 is also expressed in atherosclerotic plaque; PCSK9 released by vascular smooth muscle cells reduces LDLR expression and thus prevents the uptake of LDL cholesterol, which is associated with lipid accumulation, oxidation, and plaque formation (46). Furthermore, some studies have demonstrated that the development of atherosclerosis by PCSK9 also correlates with platelet activation, blood pressure regulation and glucose metabolism (29, 47, 48). In view of the aforementioned functional diversity of PCSK9, it is rational to consider its circulating level as a potential atherogenic risk marker for CV events.

Incongruent results were yielded in previous meta-analyses when stratifying participants according to CV risk (40, 41); Vlachopoulos et al. found that high concentration of PCSK9 associated with increased risk of CV events in the general population but not in the high-risk population, while similar significant associations were observed both in low- and high-CV risk subgroups by Qiu et al. We pooled more recent articles, mostly focusing on high-CV risk patients in the pooling analysis, which reinforced the significant correlation regardless of the degree of CV risk. Additionally, CV outcome trials have already been conducted and shown that PCSK9 inhibitors effectively reduce LDL-C and MACEs in high-risk patients with atherosclerotic CVD (1, 3). However, to date, no studies have accessed whether PCSK9 inhibitors could be used for CV prevention in the general population. Moreover, several longitudinal studies suggested that higher PCSK9 concentration was associated with the development of carotid atherosclerosis in populations free of cardiovascular disease (CVD) at baseline (49, 50). Hence, it may be worth investigating the potential role of PCSK9 inhibitors for primary CV prevention.

It is known that FH is a special group of the population who has genetic mutations resulting in persistent lifelong extremely raised LDL-C levels, premature CAD and systemic atherosclerosis (51). To the best of our knowledge, the present study is the first circulating PCSK9 meta-analysis to include studies with a high percentage of FH participants. As a special part involved in a high CV risk group, a much stronger association was found in participants with FH (as mentioned above), which might partially attribute to lifelong exposure of elevated LDL-C and substantially increased risk of early atherosclerosis among these participants (47, 52). By removing two studies specifically focusing on FH participants from the meta-analysis of MACEs (28, 33), heterogeneity mildly reduced (*I*^2^ = 48.60%, *P* = 0.009, *P*_for interaction_ = 0.001). The reduced heterogeneity might also indicate the heterogeneous nature of FH population, as reported in previous studies that the predictive value of some traditional risk factors for future MACEs was different from the general population (53, 54) Of note, although PCSK9 showed the prognostic value in FH patients, further steps are still needed to confirm it in large cohorts and different ethnic population.

It has been well-elucidated that PCSK9 antibodies significantly decrease the risk of stroke in randomized trials of therapeutic PCSK9-inhibition as comparable to the effect on MACEs. Nevertheless, it remains controversial whether PCSK9 variants associates with risk of stroke (55–57). A mendelian randomization study involving 10307 IS cases and 19,326 controls of European ancestry showed a weaker effect of PCSK9 on IS risk than on coronary heart disease (CHD) risk (58). These findings indicated that the impact of PCSK9 on the risks of IS might be of more complexity; unlike homogenous phenotype in CHD, IS involves etiological heterogeneity with different subtypes (such as large artery atherosclerosis, cardioembolic embolism, and small vessel disease) (59). It was shown that the effect of life-long lower genetically determined LDL-C and PCSK9 on different etiologically distinct IS subtypes varied materially (58, 60, 61). Moreover, in the exploration of canonical pathways of the diseases, IS are linked to natural killer cell signaling pathway rather than to lipid pathways as CHD does (62, 63). We analyzed circulating PCSK9 to elucidate the relationship between PCSK9 and stroke further. However, limited numbers of studies comprising stroke as the outcome of interest included in our meta-analysis might diminish the statistical power to detect the association for stroke, and thus the result should be viewed cautiously. Large-scale and well-designed prospective population-based studies are required to investigate further whether an increased level of PCSK9 will have predictive value for stroke and its subtypes.

PCSK9 has generally been measured by ELISA immunoassay, while the concentrations varied in a wide range (40–800 ng/ml) among different ELISA techniques (64). Studies making the head-to-head comparison of the methods to investigate the differences are scarce (64). Hence, the wide variability of results would substantially limit the utility of PCSK9 measurement in clinical practice. Moreover, it should be noted that PCSK9 circulates as mature and furin-cleaved forms in the blood. Previous studies revealed that furin-cleaved PCSK9 was inactive to regulate serum LDL-C or less activity than mature form (65–67). Nevertheless, most commercial ELISA techniques used in published studies measured the total amount of PCSK9 and could not distinguish between furin-cleaved and mature forms. The correlation between PCSK9 and CV risk might be strengthened if only the mature form was measured. Further steps are still needed to standardize, assess the agreement of different assays, and improve specificity for the total and active form of PCSK9 before using it as a CV biomarker in extensive clinical practice.

In our view, the current meta-analysis has several strengths. First, it is the most comprehensive meta-analysis on this topic to date with a relatively large number of cases and participants. Finally, the risk estimates from the fully adjusted models for each study were applied in our analyses to reduce the potential of confounding. Despite these strengths, limitations of this meta-analysis should be noted. Firstly, the pooled result of PCSK9 and MACEs showed substantial heterogeneity among the included studies, which may affect the interpretation of the results. Although we conducted on stratified and sensitivity analyses to identify the sources of heterogeneity, the heterogeneity could not be fully explained. Furthermore, meta-regression techniques are limited used in the present analysis given the lack of information for many continuous factors, such as baseline LDL, high-sensitivity C-reactive protein, and the result should therefore be viewed with caution. Secondly, original studies included in the study reported the risk estimates calculated by different multivariable models, and the pooled association lost significance in the subgroup with a lower degree of confounder adjustment. On these grounds, the combined result might potentially be influenced. Thirdly, most of the included studies used the combined CV events as the outcome of interest, making it difficult to identify the risk of specific CV events including stroke and different stroke subtypes; the statistical power might be compromised, and therefore, advanced studies focusing specific CV outcomes are warranted in future research. Finally, statistical tests for detecting publication bias in pooling the effect estimates of PCSK9 and all-cause mortality and stroke may be potentially unreliable due to less than the recommended minimum number of 10 studies analyzed (68).

## Conclusion

This meta-analysis provided further evidence that high circulating PCSK9 concentration is associated with increased risk of MACEs with a linear dose-response relationship. However, available data did not suggest a significant correlation either on stroke or all-cause mortality. Our finding suggested that measurement of PCSK9 level may have the potential to improve risk stratification for medical decision and also support the result of the beneficial clinical role of PCSK9 inhibitors. To further investigate the correlations between PCSK9 concentration and stroke and mortality, additional well-designed multicenter studies with standardized methodologies are warranted.

## Data Availability Statement

The original contributions presented in the study are included in the article/Supplementary Material, further inquiries can be directed to the corresponding author/s.

## Author Contributions

YZ had the idea for the study, did the statistical analysis with guidance from ML and WC, and drafted the manuscript. YZ and YW contributed to the study designed. YZ and WC participated in the search, data collection, and extraction. ML and WC did the major revision. All authors read and approved the final manuscript.

## Conflict of Interest

The authors declare that the research was conducted in the absence of any commercial or financial relationships that could be construed as a potential conflict of interest.
