# Supplementary material for: Association Between Circulating Proprotein Convertase Subtilisin/Kexin Type 9 and Major Adverse Cardiovascular Events, Stroke, and All-Cause Mortality: Systemic Review and Meta-Analysis
Source: Front Cardiovasc Med. 2021 Mar 2;8:617249. doi: 10.3389/fcvm.2021.617249 (PMC7960648; doi:10.3389/fcvm.2021.617249)
Supplement: Supplementary file 1 [file Data_Sheet_1.docx]

Supplementary Material

# Supplementary Data

1. **Search strategy**
   1. **Search strategy for Pubmed**

#1 "Proprotein Convertase 9"[Mesh]

#2 (((((((((((((((proprotein convertase 9[Text Word]) OR convertase 9, proprotein[Text Word]) OR proprotein convertase, subtilisin-kexin type 9[Text Word]) OR proprotein convertase, subtilisin kexin type 9[Text Word]) OR proprotein convertase subtilisin/kexin type 9[Text Word]) OR pro-protein convertase subtilisin-kexin type 9[Text Word]) OR proprotein convertase subtilisin kexin 9[Text Word]) OR PCSK9[Text Word]) OR PCSK9 protein[Text Word]) OR protein PCSK9[Text Word]) OR neural apoptosis-regulated convertase 1[Text Word]) OR neural apoptosis regulated convertase 1[Text Word]) OR NARC-1 protein[Text Word]) OR NARC 1 protein[Text Word]) OR NARC-1[Text Word])))

#3 #1 OR #2

#4 ((("Cardiovascular Diseases"[Mesh]) OR "Cerebrovascular Disorders"[Mesh]) OR "Mortality"[Mesh]) OR "Death"[Mesh]

#5 ((((((((((((((((((((((((((((((((("cardiovascular disease"[Text Word]) OR "CVD"[Text Word]) OR "cardiovascular risk"[Text Word]) OR "cardiovascular events"[Text Word]) OR "acute coronary syndromes"[Text Word]) OR "ACS"[Text Word]) OR "coronary artery disease"[Text Word]) OR "CAD"[Text Word]) OR "coronary heart disease"[Text Word]) OR "CHD"[Text Word]) OR "ischemic heart disease"[Text Word]) OR "ischemic heart disease"[Text Word]) OR "myocardial infarction"[Text Word]) OR "MI"[Text Word]) OR "myocardial ischemia"[Text Word]) OR "myocardial ischaemia"[Text Word]) OR "coronary stenosis"[Text Word]) OR "coronary restenosis"[Text Word]) OR "heart failure"[Text Word]) OR "cerebrovascular disease"[Text Word]) OR "cerebrovascular disorder"[Text Word]) OR "stroke"[Text Word]) OR "cerebral infarction"[Text Word]) OR "intracranial arteriosclerosis"[Text Word]) OR "brain ischemia"[Text Word]) OR "transient ischemic attacks"[Text Word]) OR "TIA"[Text Word]) OR "intracranial hemorrhage"[Text Word]) OR "hemorrhagic stroke"[Text Word]) OR "death" [Text Word]) OR "all-cause death"[Text Word]) OR mortality[Text Word]) OR "all-cause mortality"[Text Word]) OR "cardiovascular mortality"[Text Word]

#6 #4 OR #5

#7 “epidemiologic studies”[MeSH Terms] OR “cohort studies”[MeSH Terms]

#8 ((((((epidemiologic[Text Word]) OR cohort[Text Word]) OR longitudinal[Text Word]) OR “follow up”[Text Word]) OR observational[Text Word]) OR prospective[Text Word])))

#9 #7 OR #8

#10 #3 AND #6 AND #9

- 1. **Search strategy for Embase**

#1. 'proprotein convertase 9'/exp

#2. 'proprotein convertase 9':ab,ti OR 'convertase 9, proprotein':ab,ti OR 'proprotein convertase, subtilisin-kexin type 9':ab,ti OR 'proprotein convertase, subtilisin kexin type 9':ab,ti OR 'proprotein convertase subtilisin/kexin type 9':ab,ti OR 'pro-protein convertase subtilisin-kexin type 9':ab,ti OR 'proprotein convertase subtilisin kexin 9':ab,ti OR 'pcsk9':ab,ti OR 'pcsk9 protein':ab,ti OR 'protein pcsk9':ab,ti OR 'neural

apoptosis-regulated convertase 1':ab,ti OR 'neural apoptosis regulated convertase 1':ab,ti OR 'narc-1 protein':ab,ti OR 'narc 1 protein':ab,ti OR 'narc-1':ab,ti

#3. #1 OR #2

#4. 'cardiovascular disease'/exp

#5. 'cerebrovascular disease'/exp

#6. 'mortality'/exp

#7. 'death'/exp

#8. 'cardiovascular disease':ab,ti OR 'cvd':ab,ti OR 'cardiovascular risk':ab,ti OR 'cardiovascular events':ab,ti OR 'acute coronary syndromes':ab,ti OR 'acs':ab,ti OR 'coronary artery disease':ab,ti OR 'cad':ab,ti OR 'coronary heart disease':ab,ti OR 'chd':ab,ti OR 'ischemic heart disease':ab,ti OR 'ischaemic heart disease':ab,ti OR 'myocardial infarction':ab,ti OR 'mi':ab,ti OR 'myocardial ischemia':ab,ti OR 'myocardial ischaemia':ab,ti OR 'coronary stenosis':ab,ti OR 'coronary restenosis':ab,ti OR 'heart failure':ab,ti OR 'cerebrovascular disease':ab,ti OR 'cerebrovascular disorder':ab,ti OR 'stroke':ab,ti OR 'cerebral infarction':ab,ti OR 'intracranial arteriosclerosis':ab,ti OR 'brain ischemia':ab,ti OR 'transient ischemic attacks':ab,ti OR 'tia':ab,ti OR 'intracranial hemorrhage':ab,ti OR 'hemorrhagic stroke':ab,ti OR 'death':ab,ti OR 'all-cause death':ab,ti OR 'mortality':ab,ti OR 'all-cause mortality':ab,ti

OR 'cardiovascular mortality':ab,ti

#9 #4 OR #5 OR #6 OR #7 OR #8

#10. 'prospective study'/exp

#11. 'cohort analysis'/exp

#12. 'cohort analysis':ab,ti OR 'cohort study':ab,ti OR 'longitudinal':ab,ti OR 'follow up':ab,ti OR 'observational':ab,ti OR 'prospective':ab,ti OR 'prospective study':ab,ti

#13 #10 OR #11 OR #12

#14 #3 AND #9 AND #13

- 1. **Search strategy for Cochrane**

#1 MeSH descriptor: [Proprotein Convertase 9] explode all trees

#2 (proprotein convertase 9):ab,ti,kw OR (convertase 9, proprotein):ab,ti,kw OR (proprotein convertase, subtilisin-kexin type 9):ab,ti,kw OR (proprotein convertase, subtilisin kexin type 9):ab,ti,kw OR (pro-protein convertase subtilisin-kexin type 9):ab,ti,kw OR (proprotein convertase subtilisin kexin 9):ab,ti,kw OR (PCSK9):ab,ti,kw OR (PCSK9 protein):ab,ti,kw OR (protein PCSK9):ab,ti,kw OR (neural apoptosis-regulated convertase 1):ab,ti,kw OR (neural apoptosis regulated convertase 1):ab,ti,kw OR (NARC-1 protein):ab,ti,kw OR (NARC 1 protein):ab,ti,kw OR (NARC-1):ab,ti,kw

#3 #1 or #2

#4 MeSH descriptor: [Cardiovascular Diseases] explode all trees

#5 MeSH descriptor: [Cerebrovascular Disorders] explode all trees

#6 MeSH descriptor: [Mortality] explode all trees

#7 MeSH descriptor: [Death] explode all trees

#8 #4 or #5 or #6 or #7

#9 (cardiovascular disease):ab,ti,kw OR (CVD):ab,ti,kw OR (cardiovascular risk):ab,ti,kw OR (cardiovascular events):ab,ti,kw OR (acute coronary syndromes ):ab,ti,kw OR (ACS):ab,ti,kw OR (coronary artery disease ):ab,ti,kw OR (CAD):ab,ti,kw OR (coronary heart disease ):ab,ti,kw OR (CHD):ab,ti,kw OR (ischemic heart disease):ab,ti,kw OR (ischaemic heart disease):ab,ti,kw OR (myocardial infarction ):ab,ti,kw OR (MI):ab,ti,kw OR (myocardial ischemia ):ab,ti,kw OR (myocardial ischaemia):ab,ti,kw OR (coronary stenosis):ab,ti,kw OR (coronary restenosis):ab,ti,kw OR (heart failure):ab,ti,kw OR (cerebrovascular disease):ab,ti,kw OR (cerebrovascular disorder):ab,ti,kw OR (stroke):ab,ti,kw OR (cerebral infarction):ab,ti,kw OR (intracranial arteriosclerosis):ab,ti,kw OR (brain ischemia):ab,ti,kw OR (transient ischemic attacks):ab,ti,kw OR (TIA):ab,ti,kw OR (intracranial hemorrhage):ab,ti,kw OR (hemorrhagic stroke):ab,ti,kw OR (death):ab,ti,kw OR (all-cause death):ab,ti,kw OR (mortality):ab,ti,kw OR (all-cause mortality):ab,ti,kw OR (cardiovascular mortality):ab,ti,kw

#10 #8 or #9

#11 MeSH descriptor: [Epidemiologic Studies] explode all trees

#12 MeSH descriptor: [Cohort Studies] explode all trees

#13 #11 or #12

#14 (epidemiologic):ab,ti,kw OR (cohort):ab,ti,kw OR (longitudinal):ab,ti,kw OR (follow-up):ab,ti,kw OR (observational):ab,ti,kw OR (prospective):ab,ti,kw

#15 #13 or #14

#16 #3 and #10 and #15

**Supplementary Tables**

1. **Table S1. MOOSE checklist**

| **Criteria** | **Comments of how the criteria were handled in the meta-analysis** |
| --- | --- |
|  | Reporting of background should include |
| Problem definition | Proprotein convertase subtilisin/kexin type 9 (PCSK9), a pivotal protein in low-density lipoprotein cholesterol (LDL-C) metabolism, has been validated to be an established target for LDL-C lowing and cardiovascular events (CVEs) reduction. Nevertheless, PCSK9 in relation to CVEs and all-cause mortality have yielded inconsistent results. Therefore, the potential predictive ability of circulating levels of PCSK9 remains to be summarized quantitatively. |
| Hypothesis statement | Higher circulating concentration of PCSK9 was associated with increased risk of major adverse cardiovascular events (MACEs), stroke and all-cause mortality. |
| Description of study outcomes | MACEs, stroke, and all-cause mortality. MACEs were defined as composite outcomes, including fatal and non-fatal coronary artery disease (CAD), fatal and non-fatal stroke, and heart failure. |
| Type of exposure or intervention used | Circulating concentration of PCSK9 |
| Type of study designs used | ﻿Prospective cohort study or ﻿nested case-control study within prospective cohort. |
| Study population | Adults of any age across different countries |
|  | Reporting of search strategy should include |
| Qualifications of searchers | Two trained reviewers are indicated in the author list. Disagreement between the two reviewers was resolved by consensus. |
| Search strategy, including time period included in the synthesis and keywords | We searched electronic databases (PubMed, Embase, and Cochrane) up to July 2020 using a combined MeSH heading and key word search strategy; The query syntax of searching was shown in Supplementary material-Search strategy. |
| Databases and registries searched | PubMed, Embase and Cochrane Library database were searched, and we also checked the reference lists to identify studies that might have been missed. |
| Search software used, name and version, including special features | No search software was applied. NoteExpress was used to merge retrieved citations and eliminate duplications. |
| Use of hand searching | To avoid missing any relevant study, we also checked and manually searched the references of the included articles. |
| List of citations located and those excluded, including justifications | All steps and details of the literature search process were outlined in the flow chart (Figure 1). |
| Method of addressing articles published in languages other than English | Our search had no restriction to language. |
| Method of handling abstracts and unpublished studies | Abstract, and unpublished studies were excluded. |
| Description of any contact with authors | We did not contact authors for the detailed information of primary studies and unpublished studies. |
|  | Reporting of methods should include |
| Description of relevance or appropriateness of studies assembled for assessing the hypothesis to be tested | Detailed inclusion criteria were described in the methods section. |
| Rationale for the selection and coding of data | Data extracted from each of the studies were relevant to type and amount of participants, study type, proportion of men, mean age, duration of follow-up, number of outcome events, measurement method of PCSK9, PCSK9 concentration, adjusted confounding factors, and the most fully-adjusted risk estimate with 95% CIs, etc. |
| Documentation of how data were classified and coded (eg, multiple raters, blinding, and interrater reliability) | Study characteristics and relevant data were extracted independently by two investigators. |
| Assessment of confounding | Restricted the analysis to multivariable-adjusted risk estimates of each included articles. Subgroup analysis was conducted by different degree of confounding factors adjustment. |
| Assessment of study quality, including blinding of quality assessors; stratification or regression on possible predictors of study results | The Newcastle Ottawa Scale (NOS) was used to assess the quality of the included studies. Subgroup and meta-regression analyses were performed. |
| Assessment of heterogeneity | Cochran’s Q test and I^2^ statistic were calculated to quantify heterogeneity among studies. Subgroup analyses and meta-regression were performed according to study characteristics to explore the source of heterogeneity. |
| Description of statistical methods in sufficient detail to be replicated | Description of methods for meta-analyses, subgroup analysis, sensitivity analysis, and assessment of publication bias were detailed in the methods section. |
| Provision of appropriate tables and graphics | In our manuscript: one tables demonstrate studies characteristics; seven graphics present the flow chart, forest plots of MACEs and all-cause mortality, subgroup analyses results, dose-response linear relationship and funnel plots exploring publication bias. Supplementary materials provide graphics for sensitivity analyses and forest plots of stroke. |
|  | Reporting of results should include |
| Graph summarizing individual study estimates and overall estimate | Figure 2,5, supplementary material Figure S2 |
| Table giving descriptive information for each study included | See detailed characteristics of the included studies (Table 1) |
| Results of sensitivity testing | See results of sensitivity analysis (Supplementary file Figure S1) |
| Indication of statistical uncertainty of findings | 95% confidence intervals, I^2^ values, results of sensitivity analyses, and publication analysis. |
|  | Reporting of discussion should include |
| Quantitative assessment of bias | We discussed the reason for not detecting publication bias in pooling the effect estimates of PCSK9 and all-cause death and stroke. |
| Assessment of quality of included studies | We discussed the results of the subgroup and meta-regression analyses, and potential reasons for the observed heterogeneity. |
|  | Reporting of conclusions should include |
| Consideration of alternative explanations for observed results | Each original study reported the risk estimate calculated by different multivariable model, and the pooled association lost significance in subgroup with a lower degree of confounder adjustment in analyzed studies. On these grounds, the combined result might potentially be influenced, though the fully adjusted models for each study were used in our analyses. |
| Generalization of the conclusions | Our finding suggested that measurement of PCSK9 level might have the potential to improve risk stratification for medical decision and also support the result of beneficial clinical role of PCSK9 inhibitors. |
| Guidelines for future research | Additional well-designed multicenter studies with standardized methodologies are needed to evaluate the correlation between PCSK9 concentration and stroke. |
| Disclosure of funding source | See funding section. |

## Supplementary Figures

1. **Figure S1. ﻿Sensitivity test of single study on combined effect estimation of major adverse cardiovascular events**

**
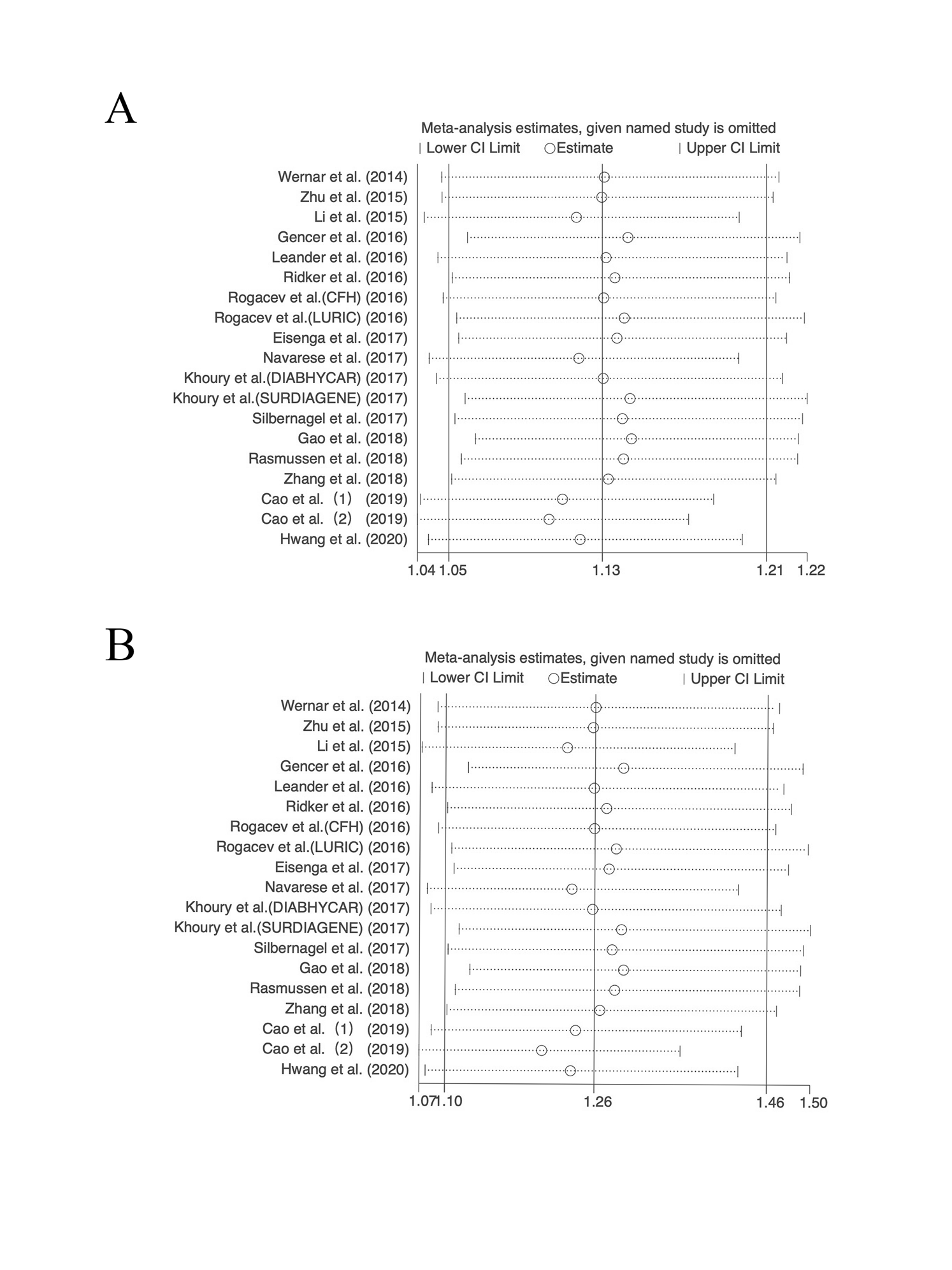
**

(A) Per one standard derivation increase in baseline proprotein convertase subtilisin/kexin type 9 levels, (B) top versus bottom tertile of baseline proprotein convertase subtilisin/kexin type 9.

CFH indicates Cardiovascular and Renal Outcome in CKD 2–4 Patients—The Forth Homburg evaluation; LURIC, Ludwigshafen Risk and Cardiovascular Health Study; DIABHYCAR, Non-Insulin Dependent Diabetes, Hypertension, ﻿Microalbuminuria or Proteinuria, Cardiovascular Events and Ramipril; SURDIAGENE, Survie, Diabète de type 2 et Génétique; CI, confidence intervals.

1. **Figure S2. Associations between circulating proprotein convertase subtilisin/kexin type 9 and risk of stroke**

**
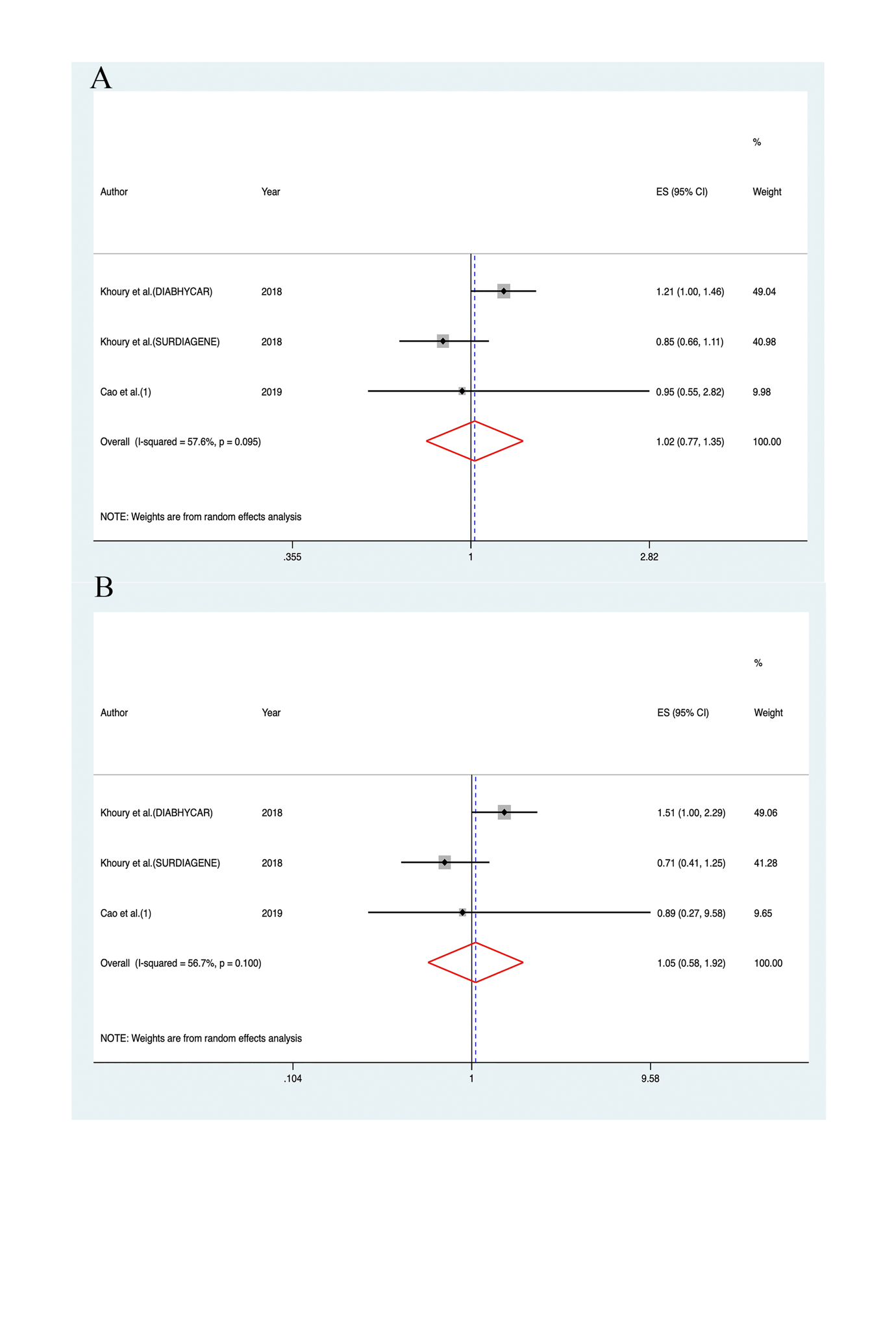
**

1. Per one standard derivation increase in baseline proprotein convertase subtilisin/kexin type 9 levels, (B) top versus bottom tertile of baseline proprotein convertase subtilisin/kexin type 9.

DIABHYCAR indicates Non-Insulin Dependent Diabetes, Hypertension, ﻿Microalbuminuria or Proteinuria, Cardiovascular Events and Ramipril; SURDIAGENE, Survie, Diabète de type 2 et Génétique; ES, effect size; CI, confidence intervals.
